# Habitual Sleep Duration and the Colonic Mucosa-Associated Gut Microbiota in Humans—A Pilot Study

**DOI:** 10.3390/clockssleep3030025

**Published:** 2021-07-01

**Authors:** Ritwick Agrawal, Nadim J. Ajami, Sonal Malhotra, Liang Chen, Donna L. White, Amir Sharafkhaneh, Kristi L. Hoffman, David Y. Graham, Hashem B. El-Serag, Joseph F. Petrosino, Li Jiao

**Affiliations:** 1 Section of Pulmonary, Critical Care and Sleep Medicine, Michael E. DeBakey VA Medical Center, Houston, TX 77030, USA; Ritwick.Agrawal@bcm.edu (R.A.); amirs@bcm.edu (A.S.); 2 The Alkek Center for Metagenomics and Microbiome Research, Department of Molecular Virology and Microbiology, Baylor College of Medicine, Houston, TX 77030, USA; nadim.ajami@gmail.com (N.J.A.); Kristi.hoffman@bcm.edu (K.L.H.); jpetrosi@bcm.edu (J.F.P.); 3 Department of Pediatrics, Texas Children Hospital, Houston, TX 77030, USA; sonal.malhotra@bcm.edu; 4 Department of Medicine, Baylor College of Medicine, Houston, TX 77030, USA; Liangc@bcm.edu (L.C.); dwhite1@bcm.edu (D.L.W.); dgraham@bcm.edu (D.Y.G.); hasheme@bcm.edu (H.B.E.-S.); 5 Center for Innovations in Quality, Effectiveness and Safety, Michael E. DeBakey VA Medical Center, Houston, TX 77030, USA; 6 Texas Medical Center Digestive Disease Center, Houston, TX 77030, USA; 7 Dan L Duncan Comprehensive Cancer Center, Baylor College of Medicine, Houston, TX 77030, USA; 8 Center for Translational Research on Inflammatory Diseases, Michael E. DeBakey VA Medical Center, Houston, TX 77030, USA; 9 Section of Gastroenterology, Michael E. DeBakey VA Medical Center, Houston, TX 77030, USA

**Keywords:** sleep, gut microbiome, circadian rhythm, diet, *Sutterella*

## Abstract

We examined the association between the colonic adherent microbiota and nocturnal sleep duration in humans. In a cross-sectional study, 63 polyp-free adults underwent a colonoscopy and donated 206 mucosal biopsies. The gut microbiota was profiled using the 16S rRNA gene sequencing targeting the V4 region. The sequence reads were processed using UPARSE and DADA2, respectively. Lifestyle factors, including sleep habits, were obtained using an interviewer-administered questionnaire. We categorized the participants into short sleepers (<6 h per night; *n* = 16) and normal sleepers (6–8 h per night; *n* = 47) based on self-reported data. Differences in bacterial biodiversity and the taxonomic relative abundance were compared between short vs. normal sleepers, followed by multivariable analysis. A false discovery rate-adjusted *p* value (*q* value) < 0.05 indicated statistical significance. The bacterial community composition differed in short and normal sleepers. The relative abundance of *Sutterella* was significantly lower (0.38% vs. 1.25%) and that of *Pseudomonas* was significantly higher (0.14% vs. 0.08%) in short sleepers than in normal sleepers (*q* values < 0.01). The difference was confirmed in the multivariable analysis. Nocturnal sleep duration was associated with the bacterial community composition and structure in the colonic gut microbiota in adults.

## 1. Introduction

In modern society, nearly one-third of adults sleep for less than six hours daily [1]. Chronic sleep deprivation has been associated with adverse health outcomes, including cardiovascular morbidity, immunosuppression, obesity, type 2 diabetes, cancer, and increased all-cause mortality [2]. Alteration in fecal gut microbiota, i.e., dysbiosis, has also been associated with these diseases [3]. Specifically, our previous study showed that short or long sleep duration was associated with an increased risk of colorectal cancer [4]. Diet and dysbiosis are consistently shown to be associated with the risk of colorectal cancer [5]. Therefore, the link between sleep phenotype and gut microbiota is biologically plausible.

Because sleep deprivation and gut microbiota have been associated with neuropsychological conditions, including depression [6], dementia [7], Parkinsonism [8,9], and autism spectrum disorder [10,11], the role of bi-directional signaling between the gut microbiota and the brain has been suggested [12]. Several mechanisms have been proposed in this bi-directional relation, including the vagus nerve and the hypothalamic–pituitary–adrenal axis, regulation of homeostasis and inflammation, and modulation of brain function and behavior [13]. Furthermore, neurotransmitters and metabolites produced by microbes, including tryptophan, melatonin, serotonin, gamma-aminobutyric acid (GABA), and short-chain fatty acids, can also impact this bi-directional relation [14]. In addition, both sleep and gut microbiota show circadian rhythms [15]. Therefore, it is plausible that sleep is intrinsically associated with gut microbiota.

Experimental studies have investigated the effects of sleep deprivation on fecal microbiota. A study in mice reported a decrease in the Lactobacillaceae family after fragmented sleep for four weeks [16]. In humans, Benedict et al. observed an increased Firmicutes:Bacteroidetes ratio following two nights of partially deprived sleep [17]. However, a cross-species study of rats and humans found that acute sleep deprivation (four hours sleep for five nights) did not significantly alter fecal microbial populations in humans. Nevertheless, a single operational taxonomic unit (OTU), TM7-3a, was increased in sleep-deprived rats [18].

A few observational studies have examined the association between fecal gut microbiota and sleep duration [19,20,21]. With no more than 40 study participants for each, these studies found differences in Bacteroidetes, Firmicutes, Lachnospiraceae, or *Blautia* between short and normal sleepers. So far, the association between colonic adherent gut microbiota and sleep phenotype has not been well-examined. However, it is well known that fecal microbiota and colonic microbiota are different [22].

Therefore, we investigated the association between sleep duration and colonic microbiota in adults with an endoscopically normal colon in this pilot study. We hypothesized that the community composition and structure of gut microbiota differed in short sleepers vs. normal sleepers.

## 2. Materials and Methods

### 2.1. Study Participants

We conducted a cross-sectional study of 69 polyp-free veterans found to have a normal colon after a colonoscopy. The detailed procedure and the exclusion criteria have been described previously [23]. For example, those patients who had antibiotic use in the past three months or had a history of cancer or inflammatory bowel disease were excluded. In addition, all participants stopped non-essential medications one week before and stopped anti-diabetic medications one day before the procedure. The Institutional Review Boards of Baylor College of Medicine and Michael E. DeBakey VA Medical Center approved the research protocol.

### 2.2. Data Collection

Approximately two weeks before the colonoscopy, informed consent form was obtained from participants when they attended the pre-screening section. We used an interviewer-administered structured questionnaire to collect demographics, lifestyle factors, medical history, and sleep habits. A total of 69 participants answered the question, “How many hours do you typically sleep at night in a 24-h period during a weekday and weekend in the last 12 months?”. The weighted average sleep hour (5/7 × weekday hour + 2/7 × weekend hour) was calculated. Six participants who reported 9 or more h of sleep daily were excluded from the analysis. Participants were then categorized into 47 normal sleepers (6–8 h) vs. 16 short sleepers (<6 h). A total of 39 participants also completed the self-administered Block food frequency questionnaire (FFQ, 2005 version) [24]. The healthy eating index (HEI) 2005 was calculated [25].

### 2.3. Tissue Collection and DNA Extraction

The complete colonoscopy was performed under conscious sedation. We collected 206 mucosal biopsies (cecum, ascending, transverse, descending, and sigmoid colon or rectum) from 63 participants when feasible. All biopsies were collected between 9:00 a.m. to 2:00 p.m. daily and then immediately placed in a sterile tube (RNase- and DNase-free) on dry ice. The samples were transferred to a −80 °C freezer within 15 min until use. Microbial genomic DNA was extracted using the MO BIO PowerLyzer UltraClean Tissue & Cell (MO BIO Laboratories, Carlsbad, CA, USA). All DNA samples were stored at −80 °C until further analysis.

### 2.4. Library Construction, 16S rRNA Sequencing, and Bioinformatics

The 16S rRNA gene hypervariable region 4 (V4) was amplified and sequenced on the MiSeq platform (Illumina, San Diego, CA, USA) using the 2 × 250 bp paired-end protocol [26]. Sequences were assigned into operational taxonomic units (OTUs) at a similarity of 97% using the SILVA v128 reference database and the UPARSE algorithm [27]. In addition, the sequence reads were processed, aligned, and categorized using the Divisive Amplicon Denoising Algorithm 2 (DADA2) v1.10.1 package in R v3.3.3 [28]. The DADA2 described the microbial communities using the unique amplicon taxonomic variants (ASVs). Filtered reads were de-replicated and de-noised using the DADA2 default parameters. After building the ASV table and removing chimeras, taxonomy was assigned using the SILVA 132 SSU NR99 database.

### 2.5. Statistical Analysis

The differences in characteristics between short vs. normal sleepers were evaluated using Student’s test or Fisher’s exact test when appropriate. The Agile Toolkit for Incisive Microbial Analyses (ATIMA) was used for analyzing the microbiota data. The alpha-diversity of the OTUs or ASVs and the relative abundance of the taxa between short vs. normal sleepers were compared using Wilcoxon signed-rank test. We used the PERMANOVA to test the beta-diversity (the bacterial community composition) of the OTUs/ASVs and used the principal coordinate analysis (PCoA) plots to visualize the dissimilarity of the community composition using the weighted Bray–Curtis as the distance matrix.

For those bacterial genera (relative abundance > 1%) that differed by sleep duration, we used a multivariable negative binomial regression model to obtain the incidence rate ratio (IRR) and its 95% confidence interval (CI) of having a non-zero bacterial count in short vs. normal sleepers. The covariates included age, ethnicity (non-Hispanic white, African Americans, and Hispanics), body mass index (BMI), cigarette smoking (yes vs. no), alcohol use (never, former, and current), hypertension, and type 2 diabetes (yes vs. no). To account for multiple colon biopsies taken from 38 of 63 participants, we used the panel data regression function (random effect) in STATA, treating each participant as a panel. We further included HEI scores in the model using data of 39 participants who completed the FFQ. In the sensitivity analyses, we used the biopsies from a single segment to examine the association. Lastly, we examined the association between gut microbiota using either weekday sleep hours or weekend sleep hours.

All statistical analyses were performed using the R statistical software (version 3.4.4, R foundation) or STATA 16.0 (Stata cooperation, College Station, TX, USA). All tests were two-sided. A *p* value < 0.05 indicated statistical significance. The false discovery rate (FDR)-adjusted *p* value (*q* value) was used to address multiple testing in microbiota analysis.

## 3. Results

### 3.1. General Characteristics of Study Participants

Table 1 shows that there were no statistically significant differences in the demographics between normal and short sleepers. Normal sleepers had a higher proportion of diabetes than short sleepers. There was no significant difference in the composition of gut microbiota by colon segment (Supplementary Figure S1). Nevertheless, a similar proportion of biopsies was obtained from each segment in normal and short sleepers. A similar proportion of biopsies was collected in the same season in normal and short sleepers.

### 3.2. Biodiversity

**Figure 1:**
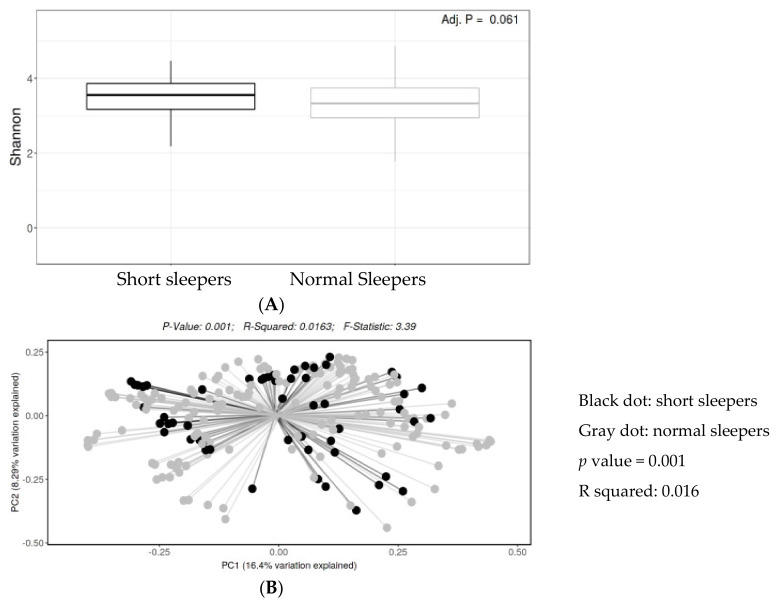
(**A**) Boxplot of the Shannon index (bacterial alpha diversity) by sleep hours (<6, short sleepers vs. 6–8, normal sleepers). There was no significant difference in bacterial richness and evenness (the Shannon index) (*q* value = 0.061, Kruskal–Wallis test) between the two groups. (**B**) Principal coordinate analysis (PCoA) with weighted Bray–Curtis dissimilarity shows the bacterial beta diversity differed significantly between the two groups (*p* value = 0.0001, PERMANOVA test). The centroids of the two groups did not overlap. The fraction of diversity captured by the coordinate was shown as a percentage in the corresponding axis. PC1 and PC2 represent the top two principal coordinates that capture most of the diversity.

Illumina pair-end sequencing returned a total of 2,832,046 raw sequences for 206 samples from 63 individuals. We obtained 2201 to 43,217 sequences per sample (median: 11,366). To limit the effect of uneven sampling, we rarefied the dataset to 1648 sequences per sample for the OTU-based data, and we rarefied the dataset to 4153 sequences per sample for the ASV-based data. The rarefaction analysis showed plateauing curves for all samples, indicating that most microbial diversity of the mucosal microbiota was sufficiently captured by sequencing. In the ASV-based analysis, there was no significant difference in bacterial community richness or evenness (alpha diversity) between short sleepers vs. normal sleepers (Shannon index *p* value = 0.06) (Figure 1A). The PCoA showed that the bacterial community composition (beta diversity) differed between the two groups (*p* value = 0.006) (Figure 1B).

### 3.3. Taxonomic Frequency

In the ASV-based analysis, the relative abundance of Firmicutes was significantly lower in short sleepers than in normal sleepers (34% vs. 40%, *q* value = 0.01), and the relative abundance of Bacteroidota was non-significantly higher in short sleepers than in normal sleepers (39% vs. 36%, *q* value = 0.11). At the family level, short sleepers had a significantly lower relative abundance of Acidaminococcaceae (0.22% vs. 0.67%), Rikenellaceae (0.47% vs. 1.31%), Sutterellaceae (0.89% vs. 1.91%), Rhodospirillales (ASV0190) (0 vs. 0.09%), and Desulfovibrionaceae (1.02% vs. 1.22%) than normal sleepers (*q* values < 0.05). However, short sleepers had significantly higher levels of Pseudomonadaceae (0.07% vs. 0.06%) and Pasteurellaceae (1.89% vs. 1.11%) than normal sleepers (*q* values < 0.05). At the genus level, among 73 genera with relative abundance > 0.05%, the relative abundance of seven bacteria was lower, and that of two bacteria was higher in shorter sleepers than in normal sleepers (Table 2).

In the OTU-based analysis, compared to normal sleepers, the relative abundance of Bacteroidetes was higher in short sleepers (37.8% vs. 44.0%, *q* value = 0.01); the relative abundance of Pseudomonadaceae was higher, and that of Acidaminococcaceae, Rikenellaceae, and Alcaligenaceae family was lower in short sleepers (*q* values < 0.05). At the genus level, the relative abundance of *Lachnoclostridium*, *Sutterella*, *Bilophila*, *Phascolarctobacterium*, and *Alistipes* was significantly lower, while that of *Pseudomonas* was significantly higher in short sleepers than in normal sleepers (*q* values < 0.05) (Supplementary Table S1).

### 3.4. Multivariable Analysis

Because the ASV and OTU-based analyses provided similar results, the IRRs (95% CI) of having non-zero bacterial count in short sleepers compared to normal sleepers were shown in Table 3 using the OTU classification. Compared to normal sleepers, the incidence rate of having *Sutterella* and *Phascolarctobacterium* was significantly lower in short sleepers. The incidence rate of having *Pseudomonas* was significantly higher in short sleepers. In the models adjusted for diet using 102 mucosal samples of 39 participants, the significant associations between *Sutterella* and *Pseudomonas* and sleep duration were observed. The models for bacteria with zero median count in both categories were not concaved.

### 3.5. Sensitivity Analysis

In the sensitivity analysis using each colon segment in the ASV-based analysis, we consistently observed a lower relative abundance of *Sutterella* in short sleepers than in normal sleepers (0.55% vs. 1.28% in the cecum, 0 vs. 2.42% in the ascending colon, 0.20% vs. 1.47% in the transverse colon, 0 vs. 1.27% in the sigmoid, and 0 vs. 1.99% in the rectum (*p* values < 0.20)). Multiple members of the Lachnospiraceae family differed by sleep duration (data not shown). Furthermore, a total of 58 participants reported both weekday and weekend sleep hours, and 53 of 58 (91.3%) of participants had the same categorization of normal vs. short sleepers. We consistently observed the lower relative abundance of *Sutterella* in short sleepers than in normal sleepers when using weekend sleep hours (0.76% vs. 1.79%, *p* value = 0.06) and weekday sleep hours (0.61% vs. 1.72%, *p* value = 0.02) in data analysis.

## 4. Discussion

In this cross-sectional study, self-reported habitual nocturnal sleep duration was associated with bacterial community composition and structure in adults. The bacteria of the Bacteroidetes and Firmicutes phyla, Lachnospiraceae, Rikenellaceae, Sutterellaceae, Prevotellaceae, and Pseudomonadaceae families differed mostly between short vs. normal sleepers. The relative abundance of *Sutterella* was significantly lower, and that of *Pseudomonas* was significantly higher in short sleepers than in normal sleepers. The differences in the relative abundance of other bacterial genera were not statistically significant in the multivariable models.

The finding of the lower *Sutterella* in short sleepers was consistent in the OTU- and the ASV-based analysis and in the sensitivity analysis using the single colon segment. *Sutterella* is lower in the colonic mucosa of children with functional abdominal pain associated with autism spectrum disorder and in the feces of patients with chronic schizophrenia and multiple sclerosis [29,30,31]. *Sutterella* was also one of the bacteria with lower relative abundance in patients with depression [32]. However, the other study found that the relative abundance of *Sutterella* spp. in feces was higher in children with autism than in controls [33]. The fact that *Sutterella* has been associated with multiple psychiatric conditions indicates that this bacterium plays a role in the microbial–brain–gut axis. In addition, the members of *Sutterella* may have immunomodulatory functions. *Sutterella* spp.*,* except for *S. wadsworthensis*, may elicit Th-17 differentiation by adhering to intestinal epithelial cells [34]. Lastly, *Sutterella* is one of the tryptophan-metabolizing bacteria [35]. The role of tryptophan in the microbiome–brain axis has been increasingly recognized [36]. However, in previous feces-based studies, *Sutterella* was not associated with sleep phenotype in humans [19,20].

We found that *Pseudomonas* was significantly more abundant in short sleepers than in normal sleepers. *Pseudomonas aeruginosa (Psae)* has the pathogenic potential of disrupting mucosa and causing respiratory infections [37]. *Psae* infection has been associated with pro-inflammatory immune response in the colon in IL-10^(–/–)^ mice with chronic colitis [14]. It has been suggested that intestinal and respiratory microbiota develop simultaneously after birth. There is a constant cross-talk between these two compartments [38,39]. However, the relative abundance of *Pseudomonas* was very low in the study samples. Our findings should be confirmed by large studies. In addition, the specific species related to sleep duration could not be defined using the 16S rRNA sequencing in our study.

*Phascolarctobacterium* was less abundant in short sleepers in the univariate model but not in the multivariable model. *Phascolarctobacterium* belongs to Negativicutes of Firmicutes phylum. The levels of *Phascolarctobacterium* were lower in 26 patients with postpartum depressive disorder (PPD) compared to 16 health controls [40]. *P. faecium* uses succinate as substrate [41]. Succinate takes part in GABA synthesis and recycle [42]. The functions of gut bacteria and their associated substrates and metabolites in the colon remained to be fully investigated.

In both ASV- and OTU-based analysis, we found that many bacteria in the Lachnospiraceae family differed in short vs. normal sleepers. Leone et al. demonstrated that a high-fat diet dampened the circadian oscillation of Lachnospiraceae bacteria in mice [43]. In another study of four-week sleep fragmentation in mice, the preferential growth of highly fermentative members of Lachnospiraceae and Ruminococcaceae was observed. When gut microbiota from sleep-fragmented mice was transplanted to germ-free mice, inflammatory responses and metabolic alterations were recapitulated [16]. In previous feces-based studies, one study found a greater relative abundance of *Blautia* (a member of Lachnospiraceae) and members of Ruminococaceae in individuals who had a better sleep quality [19]. However, the other feces-based study found an inverse association between Lachnospiraceae and sleep measures [20]. Lachnospiraceae and Ruminococcaceae (phylum Firmicutes, class Clostridia) are two major anaerobes that are found in the human gastrointestinal tract and they play roles in food fermentation. The link between diet, sleep duration, and metabolism deserves further research.

Sleep deprivation can be acute/short-term or chronic/long-term. A few existing studies had a short intervention (<20 days) in animals or humans [18,44]. These studies suggested that human gut microbiota is resistant to short-term sleep deprivation. There may be a quick adaption of the gut microbiota to short-term sleep deprivation. In our study, we asked participants to report their sleep duration over the last 12 months. The difference in gut microbiota between short vs. normal sleepers was likely reflective of chronic sleep deprivation.

Our study may be the first study investigating the association between sleep duration and the mucosa-associated gut microbiota in humans. Our pilot study had several limitations. First, we could not infer causality in this cross-sectional observational study. The causes of sleep deprivation, such as behavior, insomnia, or mental health, were unknown. Second, as a side pilot project of a larger study, we only collected self-reported sleep duration data over the last 12 months. Objective sleep phenotype data should be used to reduce the likelihood of misclassification of short vs. normal sleepers in future studies. The ascertainment of chronotype, sleep phase, or meal time would improve the characterization of the biological rhythm in humans. Third, given the relatively small sample size and the lower relative abundance of the bacteria, the findings should be considered preliminary. Fouth, we excluded participants who used antibiotics in the past three months. Some studies showed that the restoration of gut microbiota varies by individuals [45,46]. Therefore, we could not exclude the possibility that the perturbed gut microbiota due to remote use of antibiotics may have affected our findings. Finally, the study participants were mostly adult men who had an endoscopically normal colon after bowel cleansing. The generalizability of the study findings to other populations may be limited.

## 5. Conclusions

In this pilot study, we showed that the community composition and structure of the mucosa-associated gut bacteria were associated with habitual sleep duration in adults. The lower relative abundance of *Sutterella* in short sleepers was consistently observed. Further whole-genome shotgun metagenomics sequencing study should determine *Sutterella* and Lachnospiraceae species that may contribute to sleep phenotype. The causal relation between diet, gut microbiota, microbial metabolites, and sleep phenotype deserves further investigation and may open a new door to managing sleep and its related health conditions.

## Figures and Tables

**Table 1:** Basic characteristics of the participants based on sleep hours per night.

Characteristics (n (%) or Mean ± Standard Deviation)	<6 h of Sleep (Short Sleepers) N = 16 55 Mucosa	6–8 h of Sleep (Normal Sleepers) N = 47 151 Mucosa	*p* Value
Average sleep hour	4.52 ± 0.87	6.89 ± 0.63	<0.0001
Age (years)	59.4 ± 7.5	62.7 ± 5.8	0.07
Men (%)	93.7	95.7	0.73
Race/ethnicity group			
Non-Hispanic white	6 (37.5)	28 (59.6)	0.19–0.09
African American	8 (50.0)	11 (23.4)	
Hispanic	2 (12.5)	8 (17.0)	
BMI ≥ 30 kg/m^2^, n (%)			
No	8 (50.0)	15 (31.9)	0.20–0.20
Yes (obese)	8 (50.0)	32 (68.1)	
Smoking Status, n (%)			
Never	9 (56.3)	13 (27.7)	0.11
Former	5 (31.2)	26 (55.3)	
Current	2 (12.5)	8 (17.0)	
Alcohol Status, n (%)			
Never	5 (31.2)	14 (29.8)	1.00
Former	4 (25.0)	13 (27.7)	
Current	7 (43.8)	20 (42.5)	
Comorbidities, (yes, n (%))			
Hypertension	10 (66.7)	31 (66.0)	0.96
Type 2 diabetes	2 (13.3)	22 (47.9)	0.02
Total HEI Score	62.9 ± 5.60	61.0 ± 10.0	0.61
Colon segment, n (%)			
Cecum	11 (20.0)	26 (17.2)	0.95
Ascending	11 (20.0)	27 (17.9)	
Transverse	8 (14.5)	21 (13.9)	
Descending	8 (14.5)	19 (12.6)	
Sigmoid	8 (14.6)	30 (20.0)	
Rectum	9 (16.4)	28 (18.4)	
Season of biopsying			
Spring	13 (27.7)	3 (18.8)	0–0.68
Summer	11 (23.4)	5 (31.2)	
Fall	7 (14.9)	4 (25.0)	
Winter	16 (34.0)	4 (25.0)	

BMI, body mass index; HEI, healthy eating index.

**Table 2:** Relative abundance (%) of the bacterial genera in short sleepers and normal sleepers.

Genus (Phylum-Family)	Short Sleepers	Normal Sleepers	*q* Values
Lower in short sleepers	Relative abundance (%)		
*Lachnoclostridium (Firmicutes-Lachnospiraceae)*	0.40	1.50	<0.0001
*Sutterella (Proteobacteria-Sutterellaceae)*	0.38	1.25	<0.0001
*Alistipes (Bacteroidetes-Rikenellaceae)*	0.48	1.30	0.006
*Bilophila (Proteobacteria-Desulfovibrionaceae)*	0.25	0.61	0.002
*Phascolarctobacterium (Firmicutes-Acidaminococcaceae)*	0.20	0.50	0.002
*UBA1819 (Firmicutes-Ruminococcaceae)*	0.03	0.13	0.006
*Paraprevotella (Bacteroidetes-Prevotellaceae)*	0.11	0.29	0.03
Higher in short sleepers			
*Pseudomonas (Proteobacteria-Pseudomonadaceae)*	0.08	0.06	0.01
*Eubacterium_siraeum (Firmicutes-Ruminococcaceae)*	0.13	0.06	0.018

**Table 3:** The incidence rate ratio (IRR) of having non-zero bacterial count in short versus normal sleepers.

*Genera*	Short Sleepers	Normal Sleepers ^a^	IRR (95% CI) ^b^	IIRR (95% CI) ^c^	IRR (95% CI) ^d^
	Median count				
*Sutterella*	0	12.9	0.30 (0.16–0.56)	0.20 (0.06–0.60)	0.08 (0.06–0.60)
*Pseudomonas*	0.46	0	3.31 (1.42–7.76)	3.51 (1.52–8.14)	6.48 (1.77–24.0)
*Phascolarctobacterium*	1.22	4.16	0.44 (0.28–0.71)	0.32 (0.13–0.79)	0.47 (0.16–1.35)
*Lachnoclostridium*	8.39	22.5	0.52 (0.36–0.74)	0.67 (0.34–1.33)	0.38 (0.14–1.00)
*Alistipes*	1.35	7.75	0.41 (0.23–0.71)	0.83 (0.32–2.19)	0.94 (0.34–2.59)
*Bilophila*	0	5.64	0.42 (0.26–0.70)	0.74 (0.25–2.16)	

CI: confidence interval; IRR: incidence rate ratio. ^a^ Normal sleepers were the reference group in the negative binomial regression model for panel data. ^b^ The model was adjusted for age. ^c^ The model was adjusted for age, ethnicity, BMI, alcohol, smoking, hypertension, diabetes, and segment. Compared to normal sleepers, the incidence rate of having the non-zero *Sutterella* count in short sleepers decreased by 80%, and the incidence rate of having the non-zero *Pseudomonas* count increased by 2.51 times. The findings of this model were reported. ^d^ The model was also adjusted for HEI scores based on 102 mucosal samples for which diet data were collected.

## Data Availability

Data available on request due to local policy on privacy.

## Supplementary Materials

The following are available online at https://www.mdpi.com/article/10.3390/clockssleep3030025/s1, Figure S1: The community composition of colonic mucosa-associated microbiota did not differ by colon segments, Table S1: Relative abundance (%) of the bacterial genera in short sleepers and normal sleepers in the OTU-based analysis.
