# Supplementary material for: Habitual Sleep Duration and the Colonic Mucosa-Associated Gut Microbiota in Humans—A Pilot Study
Source: Clocks Sleep. 2021 Jul 1;3(3):387–97. doi: 10.3390/clockssleep3030025 (PMC8293063; doi:10.3390/clockssleep3030025)
Supplement: Supplementary file 1 [file clockssleep-03-00025-s001.zip › clockssleep-1260118-supplementary.pdf]

Supplementary Materials: The following are available online at www.mdpi.com/xxx/s1,

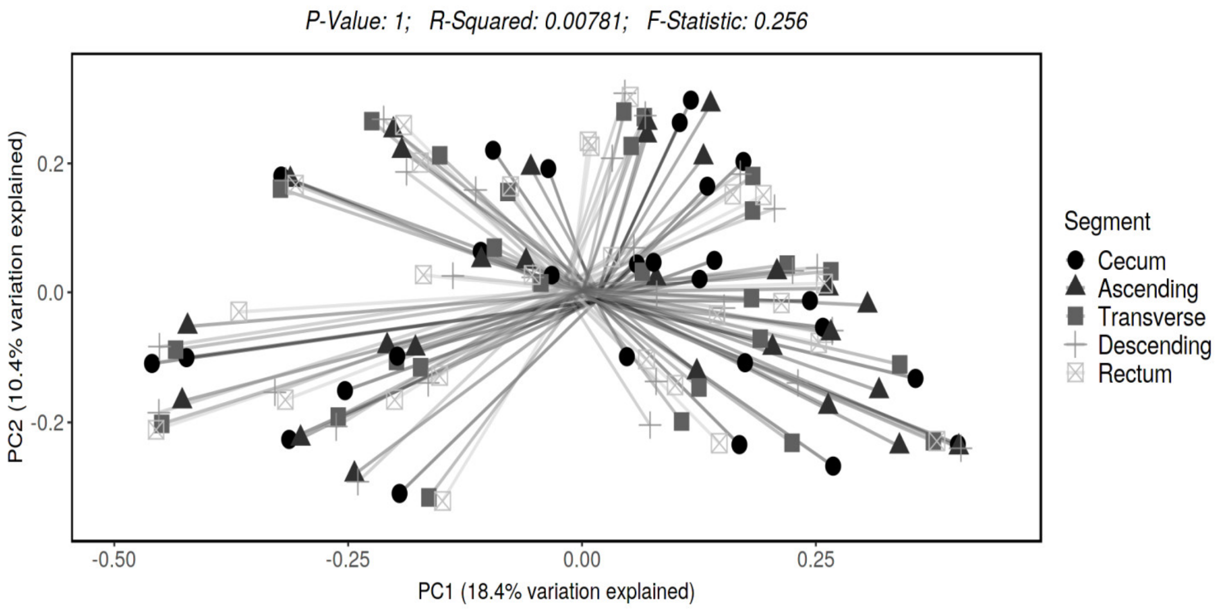

Supplemental Figure 1. The community composition of colonic mucosa-associated microbiota did not differ by colon segments.

Supplemental Table 1. Relative abundance (%) of the bacterial genera in short sleepers and normal sleepers in the OTU-based analysis

Genus (phylum-family)	Short sleepers	Normal sleepers	*q* values
Lower in short sleepers	Relative abundance (%)		
Alistipes(Bacteroidetes-Rikenellaceae)	0.57	1.54	0.019
Lachnoclostridium (Firmicutes-Lachnospiraceae)	1.06	2.01	0.005
Sutterella (Proteobacteria-Sutterellaceae)	0.38	1.25	0.00056
Bilophila (Proteobacteria-Desulfovibrionaceae)	0.27	0.63	0.0026
Paraprevotella (Bacteroidetes-Prevotellaceae)	0.06	0.28	0.017
Phascolarctobacterium(Firmicutes-Acidaminococcaceae)	0.23	0.52	0.026
Dorea (Firmicutes-Lachnospiraceae)	0.02	0.17	<0.0001
Higher in short sleepers			
Prevotellaceae(Unc00yx7)(Bacteroidetes-Prevotellaceae)	1.60	0.12	< 0.0001
Lachnospiraceae(UncO8782) (Firmicutes)	1.18	0.86	0.017
Lachnospiraceae (Unc89581) (Firmicutes)	0.50	0.19	0.0024
Lachnospiraceae (Unc01nqn) (Firmicutes)	0.40	0.11	0.005
Lachnospiraceae (Unc04i72) (Firmicutes)	0.10	0.04	0.0053
Fusicatenibacter (Firmicutes) (Firmicutes)	0.39	0.26	0.0068
Ruminococcaceae (Unc058de) (Firmicutes)	0.19	0.05	0.0068
Pseudomonas (Proteobacteria-Pseudomonadaceae)	0.14	0.08	0.0072
Lachnospiraceae (Unc02p9p) (Firmicutes)	0.13	0.06	0.018
